# Trends in Surgical Recurrence Among Pediatric Crohn’s Disease Patients Using Administrative Claims Data

**DOI:** 10.1093/crocol/otad003

**Published:** 2023-02-21

**Authors:** Matthew D Egberg, Xian Zhang, Michael Phillips, Michael D Kappelman

**Affiliations:** Department of Pediatrics, Division of Pediatric Gastroenterology, Hepatology, and Nutrition, University of North Carolina at Chapel Hill, Chapel Hill, North Carolina, USA; Department of Medicine, Division of Adult Gastroenterology, Center for Gastrointestinal Biology and Disease, University of North Carolina at Chapel Hill, Chapel Hill, North Carolina, USA; Department of Pediatrics, Division of Pediatric Gastroenterology, Hepatology, and Nutrition, University of North Carolina at Chapel Hill, Chapel Hill, North Carolina, USA; Department of Surgery, Division of Pediatric General Surgery, University of North Carolina at Chapel Hill, Chapel Hill, North Carolina, USA; Department of Pediatrics, Division of Pediatric Gastroenterology, Hepatology, and Nutrition, University of North Carolina at Chapel Hill, Chapel Hill, North Carolina, USA; Department of Medicine, Division of Adult Gastroenterology, Center for Gastrointestinal Biology and Disease, University of North Carolina at Chapel Hill, Chapel Hill, North Carolina, USA

**Keywords:** children, resection, epidemiology

## Abstract

**Background:**

Despite the growing armamentarium of medical therapies for Crohn’s disease (CD), well over half of patients with CD will require surgical intervention. We estimated the surgical recurrence risk and characterized postoperative treatment and colonoscopy use in pediatric CD patients using a large, geographically diverse administrative claims database.

**Methods:**

We analyzed postresection pediatric (≤18 years) CD patients identified in the 2007–2018 IQVIA Legacy PharMetrics administrative claims database using diagnosis and procedural codes. We estimated the surgical recurrence risk over time, characterized postoperative treatments, and reported the frequency of colonoscopy 6–15 months postoperatively.

**Results:**

Among 434 pediatric CD patients who underwent intestinal resection (median age 16 years, 46% female), risk of surgical recurrence was 3.5%, 4.6%, and 5.3% at 1, 3, and 5 years, respectively. Patients were most commonly prescribed an immune modulator (33%), anti-tumor necrosis factor agent (32%), or antibiotic (27%) postoperatively. Among 281 patients with ≥15 months of follow-up, 24% underwent colonoscopy 6–15 months postoperatively.

**Conclusions:**

Surgical recurrence risk increases over time and the low colonoscopy rates and treatment variation postoperatively represent an opportunity for practice improvement.

## Introduction

Pediatric Crohn’s disease (CD) is an immune-mediated disease of the gastrointestinal tract marked by relapsing and remitting episodes of debilitating abdominal pain, diarrhea, poor nutritional intake, and weight loss.^1^ The inflammatory process involves the full thickness of the bowel wall often leading to complications including intestinal abscesses, strictures, and fistulae. These complications require surgical management and as a result, up to 80% of patients with CD require surgical intervention during their disease course.^2,3^

Adults with CD face a high burden of postoperative disease recurrence. However, studies providing precise, generalizable estimates of surgical recurrence in the pediatric CD population are scarce, particularly in the United States. The paucity of natural history studies, randomized trials, and comparative effectiveness research poses a challenge for developing evidence-based practice guidelines regarding postoperative management in children with CD. As a result, current pediatric practice guidelines are largely rooted in expert opinion, extrapolation of adult data, or informed by data from dated, single-center studies with limited sample sizes.^4,5^ Administrative healthcare claims databases provide large sample sizes and real-world data to support generalizable estimates of pediatric surgical recurrence risk and the effectiveness of postoperative medical therapy thereby strengthening evidence-based decision making.

To that end, we utilized a large, geographically diverse administrative claims database to estimate the risk of surgical recurrence in pediatric patients with CD as well as evaluate the use of postoperative medical therapies and colonoscopy.

## Methods

### Study Design and Data Source

We conducted a retrospective cohort study of pediatric (≤18 years) patients with CD undergoing intestinal resection within the IQVIA Legacy PharMetrics Adjudicated Claims database between 2007 and 2018 to estimate rates of surgical recurrence and characterize postoperative use of medications and colonoscopy.

The IQVIA database contains longitudinal, deidentified patient-level demographics, plan enrollment information, health insurance claims for inpatient and outpatient medical services, and pharmacy prescription claims. Over 100 health plans are represented with nearly 80 million insurance plan enrollees. Prior epidemiologic studies of inflammatory bowel disease (IBD) have used the IQVIA Legacy PharMetrics Adjudicated Claims database as a representative sample of the commercially insured population in the United States.^6–11^

### Patient Selection

We analyzed all patients with ≥1 healthcare encounter with a primary diagnosis of CD identified using *International Classification of Diseases, 9th or 10th revision* (ICD-9/10) diagnosis codes (ICD-9: 555.XX; ICD-10: K50.XX). Among CD patients, we identified those with (1) ≥1 hospital admission involving an intestinal resection using *Current Procedural Terminology* (CPT) codes (Supplementary Table S1), (2) age ≤18 years on the date of the first observed intestinal resection (index surgery), and (3) ≥6 months continuous health plan enrollment prior to index intestinal resection date. We excluded patients with ≥3 medical claims, inpatient or outpatient, involving a primary ICD-9/10 diagnosis for ulcerative colitis (UC) prior to the first observed intestinal resection or ≥1 encounter with a primary ICD diagnosis code for UC in the 30 days leading up to, and including, the date of first observed intestinal resection. We excluded patients with a reasonable likelihood of UC due to significant differences in postoperative course including risk of surgical recurrence and use of immune suppressing medications. Patients with UC requiring surgical intervention undergo a multistaged total abdominal colectomy with ileal pouch anal anastomosis (TAC/IPAA) thus it is expected for patients with UC to experience “surgical recurrence” which is planned and not due to disease activity. Secondly, patients with UC undergoing TAC/IPAA are frequently considered “cured” and are not routinely managed with postoperative IBD-targeted therapies. We used a conservative definition for patients with UC in our exclusion criteria to further focus our cohort on pediatric patients with CD.

To describe the postoperative utilization of medical therapy and colonoscopy, we identified subcohorts of patients with ≥12 and ≥15 months of postoperative health plan enrollment, respectively.

### Follow-up

Follow-up began on the date of the first observed intestinal resection (index resection) and continued until whichever of the following occurred first: repeat intestinal resection, discontinuation of insurance coverage, or end of the study data.

### Outcome Measures

Our primary outcome of interest was recurrence of intestinal resection using the same CPT definitions as the index surgery. The full claims details of patients identified as having a surgical recurrence were reviewed independently by both a pediatric gastroenterologist as well as a pediatric surgeon to confirm that the surgical procedures reflected recurrence as opposed to management of surgical complications or planned follow-up procedures. Case determinations were compared between independent reviewers and any discrepancies were discussed until agreement was achieved. Surgical procedures occurring within 30 days of index resection were also reviewed and considered surgical complications and not recurrence due to CD activity, and thus not included in outcome case count.

We also characterized the postoperative treatment practices for a subcohort of pediatric patients with ≥12 months of postoperative follow-up. Medication classes included antibiotics (metronidazole and ciprofloxacin), aminosalicylates (ASA) (sulfasalazine, mesalamine, balsalazide, and olsalazine), immune modulators (thiopurines and methotrexate), steroids (prednisone, methylprednisolone, and enteral budesonide), anti-tumor necrosis factor alpha (aTNF) agents (infliximab, adalimumab, golimumab, and certolizumab), and non-aTNF biologics (natalizumab/vedolizumab and ustekinumab). We stratified the first postoperative year into 2 time periods: 0–90 days following resection and 91–365 days following resection. Prescriptions for medications between 0 and 90 days postoperatively were considered “prophylactic” treatment as clinical symptoms of disease recurrence are rare in the first 3 months following surgery. New prescriptions between 91 and 365 days postoperatively were considered “on-demand” treatment as transitioning to, or adding, therapies is most often a response to disease signs or symptoms. Prescriptions in each of these 2 time periods were mutually exclusive between periods (eg, prescriptions for mesalamine in the 91- to 365-day window do not include any patient with a prescription for mesalamine in the 0- to 90-day period).

Among patients with ≥15 months of health plan enrollment following the first observed resection, we evaluated utilization of diagnostic/noninterventional colonoscopy 6–15 months following index resection identified using CPT codes (Supplementary Table S1).

### Baseline Characteristics

We analyzed patient demographics at the start of follow-up including patient age, sex, and US census region. For the overall cohort, we characterized all CD-targeted treatment (Supplementary Table S2) prescriptions during the 6 months preceding the first observed intestinal resection. We also categorized location of intestinal resection (small bowel resection, large bowel resection, or ileocolectomy/ileocecectomy) based on CPT code. Healthcare utilization was quantified in the 6-month period leading up to the first observed intestinal resection and included the number of unique outpatient encounters, hospital admissions (excluding index resection hospitalization defined by admission within 7-day period preceding surgery date), emergency department visits, and colonoscopy.

### Statistical Analyses

We used descriptive statistics to summarize the pediatric surgical cohort at the start of follow-up. Continuous variables were reported using median and interquartile range (IQR), while categorical variables were reported using frequency count and percentage. We estimated the overall incidence rate of surgical recurrence (cases per 1000 person-years of total follow-up) at 1, 3, and 5 years postoperatively and included a 95% CI. We also described utilization of postoperative medical therapy in the 0–90 days following resection and 91–365 days following resection among patients with ≥12 months of postoperative follow-up. Lastly, we characterized the frequency of colonoscopy use in the 6–15 months following first observed resection along with median time (in days) and IQR to first colonoscopy. We used Chi2 test of trend to evaluate for a statistically significant temporal relationship in colonoscopy use. A 2-sided *P* value of ≤.05 was considered statistically significant for all comparisons. All analyses were performed using SAS version 9.4 (SAS Institute).

### Ethical Considerations

Due to the deidentified nature of the data, this study was determined to be exempt by the Institutional Review Board at the University of North Carolina at Chapel Hill.

## Results

### Cohort Characteristics

We analyzed a total of 434 pediatric patients with CD undergoing intestinal resection contained with the IQVIA Legacy PharMetrics Adjudicated Claims database between 2007 and 2018. The median age of the cohort was 16 years (IQR 14–17) with 46% females. Table 1 summarizes the demographic, preoperative treatment exposures, and healthcare utilization of the overall cohort.

**Table 1.** Characteristics of the study cohort of pediatric Crohn’s disease patients at the time of first observed intestinal resection from the IQVIA Legacy PharMetrics database 2007–2018.

Characteristic	*N*	%
Number of members	434	100
Age (years) (median, IQR)	16	(14, 17)
Sex		
Female	199	46
Region		
Northeast	102	24
Midwest	140	32
South	109	25
West	69	16
Unknown/Missing	14	3
Pay type		
Commercial	358	82
Medicaid	41	9
Self-insured	23	5
State Children’s Health Insurance Program	4	1
Unknown/Missing	8	2
Resection location (index resection)		
Small bowel only	82	19
Large bowel only	90	21
^a^Both large and small bowel	8	2
Ileocolectomy/ileocecectomy	254	59
Follow-up (days) (median, IQR)	481	(195, 1150)
Medication exposures in 6 months preoperatively		
Aminosalicylates	138	32
Antibiotics	177	41
aTNF	164	38
Immune modulators	180	41
Natalizumab/vedolizumab	4	1
Ustekinumab	2	<1
Oral or IV steroid	227	52
Budesonide	79	18
Healthcare utilization in the 6-month period prior to the first observed resection		
Any outpatient encounters	394	91
Any hospital admissions (≥7 days prior to index date)	266	61
Any ED visit	243	56
Any colonoscopies	219	50

Abbreviations: aTNF, anti-tumor necrosis factor; ED, emergency department; IQR, interquartile range; IV, intravenous.

^a^Both large and small bowel resections include independent resections of their respective anatomic locations on the date of resection and are separate from ileocolectomy/ileocecectomy.

### Incidence of Surgical Recurrence

Among the 434 pediatric patients with CD who underwent an intestinal resection, there was a total of 341 992 days of postoperative follow-up (937 patient-years). Fifteen patients (3.5%) experienced a surgical recurrence by 1 year of follow-up (incidence rate = 46/1000 patient-years; 95% CI, 27–75). The 3- and 5-year recurrence rates were 30/1000 patient-years (95% CI, 19–45) and 28/1000 patient-years (95% CI, 18–41), respectively. Cumulative risk of surgical recurrence was 3.5%, 4.6%, and 5.3% at 1, 3, and 5 years, respectively.

### Postoperative Medication Prescriptions

Of 312 patients with ≥12 months of follow-up after intestinal resection, all were prescribed ≥1 medications in the first 90 days following surgery. Twenty-one percent (21%) of the cohort were prescribed an ASA. One-third (33%) of the cohort were started on an immune modulator while 32% were prescribed an aTNF agent. Among the children prescribed antibiotics (27%) postoperatively, metronidazole was more common than ciprofloxacin (23% vs 11%). In the period ranging from 91 to 365 days postoperatively, 180 patients (58%) received ≥1 prescription for a new class of agent. Among those receiving a new agent, 15% were transitioned to, or added, an immune modulator. Anti-TNF agents were added, or transitioned to, in 13% of patients while antibiotics were prescribed to 11%, and 5% were prescribed an ASA (Table 2).

**Table 2.** Postoperative treatment prescriptions stratified by postresection time periods among 312 pediatric patients with Crohn’s disease and 12-month follow-up analyzed in the IQVIA Legacy PharMetrics database 2007–2018.

Treatment	Any use 0–90 days from surgery		Any use 91–365 days from surgery		Any use 0–365 days (total)
	*n*	%	*n*	%	*n*
Number of members	312	100	312	100	312
No pharmacologic therapy	0	—	—	—	0
Additional pharmacologic agent(s)	—	—	132	42	—
Aminosalicylates					
*Any*	66	21	16	5	82
Sulfasalazine	3	1	2	1	5
Mesalamine	61	20	13	4	74
Balsalazide	2	1	0	0	2
Olsalazine	0	0	1	0	1
Antibiotics					
*Any*	83	27	34	11	117
Metronidazole	73	23	24	8	97
Ciprofloxacin	33	11	15	5	48
Immune modulators					
*Any*	103	33	47	15	150
Thiopurine	80	26	30	10	110
Methotrexate	23	7	17	5	40
aTNF agents					
*Any*	100	32	42	13	142
Infliximab	56	18	18	6	74
Adalimumab	39	13	20	6	59
Certolizumab	6	2	4	1	10
Golimumab	0	0	0	0	0
Anti-integrin agents					
*Any*	3	1	1	0	4
Vedolizumab	3	1	1	0	4
Natalizumab	0	0	0	0	0
Anti-interleukin 12/23 agents					
Ustekinumab	2	1	3	1	5
Steroid					
Oral or IV steroid	79	25	38	12	117
Budesonide	9	3	15	5	24

Therapies are not mutually exclusive within columns but are mutually exclusive between columns. Abbreviations: aTNF, anti-tumor necrosis factor; IV, intravenous.

### Postoperative Colonoscopy Use

**Figure 1.**
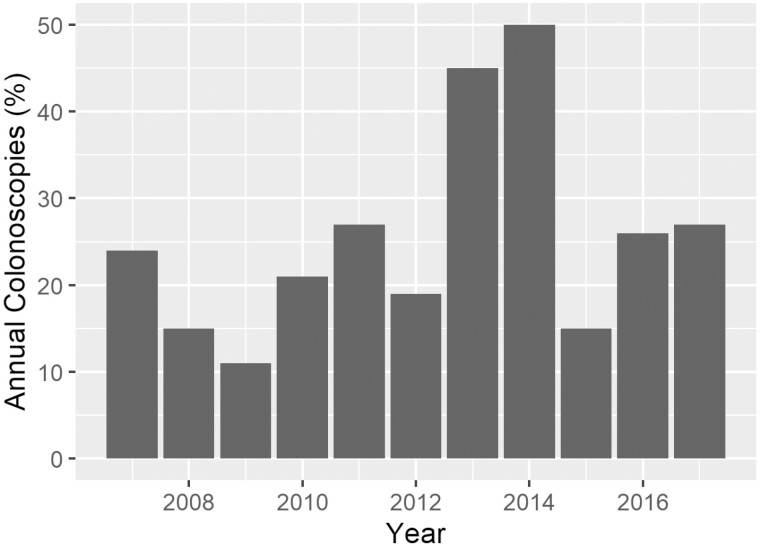
Annual frequency of diagnostic/noninterventional colonoscopy in the 6–15 months following index intestinal resection among pediatric patients with Crohn’s disease and ≥15-month follow-up in the 2007–2018 IQVIA Legacy PharMetrics Adjudicated Claims database.

Among the 281 (65% of the overall cohort) pediatric patients with CD and ≥15 months of postoperative follow-up, 67 (24%) underwent ≥1 colonoscopy between 6 and 15 months following index resection. Of those who underwent colonoscopy, the median time to first postoperative colonoscopy was 291 days (9.7 months) (IQR 233, 376). Figure 1 summarizes the use of postoperative colonoscopy over time. Despite a numerical increase in colonoscopy use over time, the temporal trend was not statistically significant (*P* = .06).

## Discussion

In this analysis of US healthcare claims from a cohort of 434 commercially insured children with CD undergoing an intestinal resection between 2007 and 2018, we observed a cumulative probability of surgical recurrence of 3.5%, 4.6%, and 5.3% at 1, 3, and 5 years postoperatively. The most prescribed postoperative treatments in the first 90 days following surgery included immune modulators, aTNF therapies, and antibiotics. Nearly 60% of the patients in the cohort utilized additional classes of CD therapies between 3 and 12 months after surgery. Importantly, only one-quarter (24%) of patients with ≥15 months of follow-up underwent colonoscopy evaluation 6–15 months following index intestinal resection without a significant temporal trend, indicating a persistent gap between actual care and recommendations from the North American Society of Pediatric Gastroenterology, Hepatology, and Nutrition (NASPGHAN) and American Gastroenterological Association (AGA).^4,12^

The risk of surgical recurrence is an important consideration in the management of postoperative pediatric CD. We observed a cumulative probability of surgical recurrence of 3.5%, 4.6%, and 5.3% at 1, 3, and 5 years, respectively. These risk estimates differ slightly from those published by Diederen et al who conducted a multicenter retrospective study of 122 children with CD undergoing first intestinal resection for refractory disease reporting a 1- and 5-year risk estimate for surgical recurrence of 2% and 12%, respectively.^13^ Important differences between the Diederen study and ours include (1) use of stricturoplasty in the definition of surgical recurrence in the Diederen study while not included in our definition thus reducing the overall sum of outcome procedures, and (2) nearly 20% of patients in the Diederen study did not receive treatment in the immediate postoperative period as compared with universal treatment of our sample. Although 1-year surgical recurrence rates were similar, early use of therapy (<90 days postoperatively) may reduce long-term recurrence rates. Our results extend prior findings through inclusion of a larger, and likely more diverse sample of pediatric patients with CD. Additionally, as this was a cohort sampled from a commercially insured US population, outcomes are reflective of more generalizable clinical care compared with the management practices of smaller studies from tertiary care centers with a handful of providers.

Diederen et al also reported a 22% risk of surgical recurrence in their cohort at 10 years postoperatively.^13^ This long-term risk elevation was underscored by a Danish National Registry study evaluating 115 pediatric patients with CD undergoing intestinal resection and demonstrated a 34% required repeat resection due to disease recurrence over a 10-year follow-up.^14^ All considered, surgical recurrence is a significant challenge for the pediatric CD population, and one that escalates over time. As such, proactive surveillance methods and effective prevention strategies must be thoughtfully developed and applied.

Postoperative disease recurrence is first detected endoscopically and is often clinically silent.^15,16^ Due to the insidious nature of postoperative recurrence and need for early intervention, proactive endoscopic surveillance is the first step to intervening on the natural history of surgical recurrence in pediatric CD. Current pediatric and adult guidelines recommend endoscopic surveillance between 6 and 12 months postoperatively.^4,12^ We characterized postoperative colonoscopy utilization using real-world data and showed that only 24% of children underwent endoscopic evaluation during the 6- to 15-month postoperative timeframe. Importantly, much of the analyzed data preceded pediatric societal recommendations for the management of postoperative CD, however, incidence of endoscopic recurrence early in the postsurgical course is well documented in the adult population.^15–18^ Thus, a similar proactive approach to endoscopic surveillance for children may increase the likelihood of detecting subclinical disease activity and promote timely treatment. Early and effective interventions may prevent, or even reverse, disease progression. The gap that exists between guideline recommendations for postoperative surveillance colonoscopy and real-world practice represents an important target for quality improvement initiatives for pediatric IBD patient care across the United States.

Timing and selection of a therapeutic approach that minimizes drug-associated toxicity and effectively prevents surgical recurrence remains a challenge. We identified a high degree of variation in the treatment prescription practices of clinicians in the first 12 months postoperatively. The heterogeneity of management underscores the need for evidence-based strategies preventing disease recurrence. Randomized controlled studies of postoperative adult CD patients suggest a role for aTNF therapies in the prevention of disease recurrence.^17,19–24^ As such, randomized comparative effectiveness trials evaluating a range of outcomes including clinical and endoscopic recurrence are needed to expand our understanding of risk/benefit tradeoffs for several candidate therapies.

An important strength of this study was the utilization of large and geographically diverse administrative claims database to provide real-world data and generalizable estimates of the natural history of postoperative pediatric CD. Data were also well suited to describe everyday postoperative care including medical treatments and the utilization of surveillance colonoscopy. Study limitations include the possibility of misclassification of the exposure and/or the outcome. To address this possibility, we attempted to minimize misclassification using conservative inclusion/exclusion criteria as well as conducted individual claim review of all cases of surgical recurrence. Second, administrative claims data are limited with respect clinical data including family history, smoking status, disease phenotype, and disease activity metrics thus unmeasured confounding is also considered. Additionally, this was a study of commercially insured pediatric patients with CD thus uninsured children are not represented. Third, median follow-up for this cohort was 24 months impacting the precision of 5-year postoperative recurrence estimates.

## Conclusions

Our study of United States commercially insured pediatric patients with CD demonstrates the nontrivial risk of postoperative recurrence in this population and suggests several ongoing knowledge gaps. Despite universal early initiation of CD treatments postoperatively, variation in postoperative medical management underscores the need for evidence-based guidance for effective recurrence prevention. Further, the markedly low frequency of postoperative colonoscopy demonstrates a gap between recommended care and everyday clinical practice. Quality improvement programs and national benchmarks targeting improved postsurgical colonoscopy rates may be effective steps in the reduction of disease recurrence and associated complications in the CD population. Lastly, more data are needed to better understand effective strategies to prevent postoperative complications including randomized comparative effectiveness trials.

## Supplementary Material

otad003_suppl_Supplementary_Table_S1

otad003_suppl_Supplementary_Table_S2

## Data Availability

The data set(s) that support the findings of this study are available from IQVIA but restrictions apply to the availability of these data which were used under license for the current study and thus are not publicly available. Data are available from the authors upon reasonable request and with permission of IQVIA.
