# Supplementary material for: Trends in Surgical Recurrence Among Pediatric Crohn’s Disease Patients Using Administrative Claims Data
Source: Crohns Colitis 360. 2023 Feb 21;5(1):otad003. doi: 10.1093/crocol/otad003 (PMC9951729; doi:10.1093/crocol/otad003)
Supplement: otad003_suppl_Supplementary_Table_S1 [file otad003_suppl_supplementary_table_s1.docx]

| **CPT** | **LARGE BOWEL Description** |
| --- | --- |
| 44140 | Colectomy, partial; with anastomosis |
| 44141 | Colectomy, partial; with skin level cecostomy or colostomy |
| 44143 | Colectomy, partial; with end colostomy and closure of distal segment (Hartmann type procedure) |
| 44144 | Colectomy, partial; with resection, with colostomy or ileostomy and creation of mucofistula |
| 44145 | Colectomy, partial; with coloproctostomy (low pelvic anastomosis) |
| 44146 | Colectomy, partial; with coloproctostomy (low pelvic anastomosis), with colostomy |
| 44147 | Colectomy, partial; abdominal and transanal approach |
| 44150 | Colectomy, total, abdominal, without proctectomy; with ileostomy or ileoproctostomy |
| 44151 | Colectomy, total, abdominal, without proctectomy; with continent ileostomy |
| 44155 | Colectomy, total, abdominal, with proctectomy; with ileostomy |
| 44156 | Colectomy, total, abdominal, with proctectomy; with continent ileostomy |
| 44157 | Colectomy, total, abdominal, with proctectomy; with ileoanal anastomosis, includes loop ileostomy, and rectal mucosectomy, when performed |
| 44158 | Colectomy, total, abdominal, with proctectomy; with ileoanal anastomosis, creation of ileal reservoir (S or J), includes loop ileostomy, and rectal mucosectomy, when performed |
| 44204 | Laparoscopy, surgical; colectomy, partial, with anastomosis |
| 44206 | Laparoscopy, surgical; colectomy, partial, with end colostomy and closure of distal segment (Hartmann type procedure) |
| 44207 | Laparoscopy, surgical; colectomy, partial, with anastomosis, with coloproctostomy (low pelvic anastomosis) |
| 44208 | Laparoscopy, surgical; colectomy, partial, with anastomosis, with coloproctostomy (low pelvic anastomosis) with colostomy |
| 44210 | Laparoscopy, surgical; colectomy, total, abdominal, without proctectomy, with ileostomy or ileoproctostomy |
| 44211 | Laparoscopy, surgical; colectomy, total, abdominal, with proctectomy, with ileoanal anastomosis, creation of ileal reservoir (S or J), with loop ileostomy, includes rectal mucosectomy, when performed |
| 44212 | Laparoscopy, surgical; colectomy, total, abdominal, with proctectomy, with ileostomy |
|  | **SMALL BOWEL Description** |
| 44120 | Enterectomy, resection of small intestine; single resection and anastomosis |
| 44121 | Enterectomy; resection of small bowel, multiple |
| 44125 | Enterectomy, resection of small intestine; with enterostomy |
| 44202 | Laparoscopy, surgical; enterectomy, resection of small intestine, single resection and anastomosis |
|  | **ILEO-CECAL Description** |
| 44160 | Colectomy, partial, with removal of terminal ileum with ileocolostomy |
| 44205 | Laparoscopy, surgical; colectomy, partial, with removal of terminal ileum with ileocolostomy |
|  | **COLONOSCOPY** |
| 45378 | Colonoscopy, flexible; diagnostic, including collection of specimen(s) by brushing or washing, when performed (separate procedure) |
| 45380 | Colonoscopy, flexible; with biopsy, single or multiple |

**Supplemental Table 1:** Current Procedural Terminology (CPT) codes used for cohort inclusion and outcomes definition.
