# Supplementary material for: Trends in Surgical Recurrence Among Pediatric Crohn’s Disease Patients Using Administrative Claims Data
Source: Crohns Colitis 360. 2023 Feb 21;5(1):otad003. doi: 10.1093/crocol/otad003 (PMC9951729; doi:10.1093/crocol/otad003)
Supplement: otad003_suppl_Supplementary_Table_S2 [file otad003_suppl_supplementary_table_s2.docx]

| **Treatment Class** | **Agents** |
| --- | --- |
| Aminosalicylates | Sulfasalazine, Mesalamine, Balsalazide, Olsalazine |
| Antibiotics | Metronidazole, Ciprofloxacin |
| Immune Modulators | Thiopurines (Azathioprine/6-mercaptopurine), Methotrexate |
| Anti-TNF Agents | Infliximab, Adalimumab, Certolizumab Pegol, Golimumab |
| Anti-Integrin Agents | Vedolizumab, Natalizumab |
| Anti-IL*-12/23 | Ustekinumab |
| Steroid | Systemic Steroids (Prednisone/(methyl)prednisolone), enteral Budesonide |

**Supplemental Table 2:** Crohn’s disease (CD) medications analyzed among post-operative pediatric patients with CD in the IQVIA Legacy PharMetrics Administrative Claims database 2007-2018. *IL = Interleukin
